# HCK maintains the self-renewal of leukaemia stem cells via CDK6 in AML

**DOI:** 10.1186/s13046-021-02007-4

**Published:** 2021-06-24

**Authors:** Zheng Li, Fangce Wang, Xiaoxue Tian, Jun Long, Bin Ling, Wenjun Zhang, Jun Xu, Aibin Liang

**Affiliations:** 1 Department of Haematology, Tongji Hospital, Tongji University School of Medicine, 1239 Siping Road, 200092 Shanghai, P.R. China; 2 The Second People’s Hospital of Yunnan Province, 650000 Kunming, P.R. China; 3 East Hospital, Tongji University School of Medicine, 1239 Siping Road, 200092 Shanghai, P.R. China

**Keywords:** Acute myeloid leukaemia (AML), Haematopoietic cell kinase (HCK), Leukaemia stem cells (LSCs), Self-renewal, Cell cycle, Cyclin-dependent kinase 6 (CDK6).

## Abstract

**Background:**

Leukaemia stem cells (LSCs) are responsible for the initiation, maintenance, and recurrence of acute myeloid leukaemia (AML), an aggressive haematological malignancy associated with drug resistance and relapse. Identifying therapeutic LSC targets is critical to curing AML.

**Methods:**

Bioinformatics databases were used to identify therapeutic LSC targets. The conditional knockout mice were used to analyse the role of HCK in leukaemogenesis or normal haematopoiesis. Colony-forming assays, cell counting, and flow cytometry were used to detect the viability and function of leukaemia cells. RT-PCR, western blotting, and RNA sequencing were used to detect mRNA and protein expression.

**Result:**

HCK is expressed at higher levels in LSCs than in haematopoietic stem cells (HSCs), and high HCK levels are correlated with reduced survival time in AML patients. Knockdown of HCK leads to cell cycle arrest, which results in a dramatic decrease in the proliferation and colony formation in human AML cell lines. Moreover, HCK is required for leukemogenesis and leukaemia maintenance in vivo and in vitro. HCK is necessary for the self-renewal of LSCs during serial transplantation and limiting dilution assay. The phenotypes resulting from HCK deficiency can be rescued by CDK6 overexpression in the human cell line. RNA sequencing and gene expression have demonstrated that HCK may sustain cell cycle entry and maintain the self-renewal ability of LSCs through activating the ERK1/2-c-Myc-CDK6 signalling axis. In contrast, HCK deletion does not affect normal haematopoiesis or haematopoietic reconstruction in mice.

**Conclusions:**

HCK maintains the self-renewal of leukaemia stem cells via CDK6 in AML and may be an ideal therapeutic target for eradicating LSCs without influencing normal haematopoiesis.

**Supplementary Information:**

The online version contains supplementary material available at 10.1186/s13046-021-02007-4.

## Background

Acute myeloid leukaemia (AML) constitutes a heterogeneous clonal malignant disease characterized by proliferative, clonal, abnormally differentiated, and occasionally poorly differentiated cells that infiltrate bone marrow (BM), peripheral blood, and other tissues [1, 2]. In the past 50 years, the treatment strategies, a combination of cytarabine and anthracycline-based regimens combined with haematopoietic stem cell transplantation, have not changed significantly [3, 4]. At present, adult AML patients who are 60 years old or younger are cured in 35 to 40 % of cases, while patients older than 60 are cured in only 5 to 15 % of cases [1]. For AML patients who achieve remission, the relapse rate is 50 %, and the 5-year overall survival rate is dismal 30 % [5–7]. New strategies to treat AML are what researchers have been looking for.

 Mounting evidence suggests that leukaemia stem cells (LSCs) are responsible for the initiation, development, and recurrence of AML [8, 9]. LSCs are defined functionally as leukaemic cells that are capable of self-renewal. LSCs self-renew to generate more LSCs and give rise to differentiated leukaemia cells [9–11]. Post-therapy after remission, consolidation, and maintenance therapy for AML aims to maintain or prolong remission by eliminating residual leukaemic cells and preventing relapse. Disappointingly, novel therapies that eradicate AML LSCs have still not reached clinical practice. Thus, studying the underlying mechanisms of how LSCs undergo self-renewal could reveal novel therapeutic strategies. LSCs are very similar to haematopoietic stem cells (HSCs) in terms of their phenotype and self-renewal ability [8]. It is critical to accurately characterize the subtle differences between HSCs and LSCs to develop LSC-specific therapies that spare HSCs [12]. Detailed functional characterization of the genetic changes that drive AML will help us understand the vital differences between HSCs and LSCs and develop LSC-specific therapies.

Haematopoietic cell kinase (HCK), a member of the Src family of nonreceptor protein tyrosine kinases (SFKs), is expressed in the myeloid cell and B-lymphocyte cell lineages [13]. Aberrant activation of HCK, a tumour cell-intrinsic oncogene, triggers haematological malignancies by physical association with oncogenic fusion proteins or functional interactions with receptor tyrosine kinases [13–17]. Deregulation of HCK levels has been detected in BM malignancies. For example, HCK exhibits increased expression in chronic myeloid leukaemia (CML), multiple myeloma (MM), and acute lymphoblastic leukaemia (ALL) [15, 16, 18]. Additionally, HCK activation has been described in solid tumours, such as breast cancer and colon cancer, and this activation is correlated with the induction of chemotherapy resistance [13]. In 2010, researchers identified that HCK expression in LSCs was higher than that in HSCs, and researchers found that an HCK inhibitor (RK-20,449) seemed to eliminate human LSCs in a mouse xenograft model in 2013 [19, 20]. However, the detailed roles of HCK in AML and normal haematopoiesis are not yet clear.

In this study, HCK was highly expressed in LSCs, and knockdown of HCK led to cell cycle arrest, which resulted in a dramatic decrease in the proliferation and colony formation of human AML cell lines. Moreover, HCK is required for leukaemogenesis and leukaemia maintenance in vivo and in vitro. HCK is necessary for the self-renewal of LSCs during serial transplantation and limiting dilution assay. In terms of mechanism, we demonstrate that HCK may activate the downstream ERK1/2 pathway and subsequently activate c-Myc and CDK6 to sustain the self-renewal ability of LSCs. In contrast, HCK deletion does not affect normal haematopoiesis or haematopoietic reconstruction in mice. In summary, HCK may be an ideal therapeutic target for eradicating LSCs without influencing normal haematopoiesis.

## Materials and methods

### Bioinformatic database mining

We performed differential gene analysis using RNA-sequence data in the GEO database (GSE63270 and GSE24797; https://www.ncbi.nlm.nih.gov/gds). The GEPIA database (http://gepia.cancer-pku.cn/) and The cBioPortal database (http://www.cbioportal.org) were applied to analyse the level of HCK mRNA expression in tumour tissues and normal tissues of AML and the correlation between the level of HCK mRNA expression and patient overall survival [21, 22]. The Cancer Cell Line Encyclopedia (CCLE) database (www.portals.broadinstitute.org/ccle) was mined to analyse the level of HCK mRNA transcripts in kinds of cell lines.

### Cell isolation

Human CD34^+^ CD38^−^ LSCs or cord blood CD34^+^ CD38^−^ were identified by antibodies against human, CD34 (BD Pharmigen, 550,761), CD38(BD Pharmigen, 562,444). Red blood cells were lysed using RBC Lysis Buffer (Solarbio, China) before staining. Stained cells were then sorted by the BD FACSAria II instrument. AML patient sample information is in Supplementary Table 1. All human samples were conducted with approval from the ethical review of biomedical research of Shanghai Tongji Hospital.

### Mice

HCK-knockout mice (denoted as HCK^−/−^) were purchased from Humangen Biotech Inc. Conditional knockout mice (exon-3 floxed; HCK ^fl/fl^) were successively mated with H11-CAG-FLPO mice (Nanjing Biomedical Research Institute of Nanjing University, China) and Rosa26-CreERT2 mice (Nanjing Biomedical Research Institute of Nanjing University, China) to generate HCK ^fl/fl^-Rosa26-CreER mice (Supplemental Fig. 1a, b). The C57BL/6-CD45.1 mouse used as transplant recipients were provided by Caiwen Duan at Shanghai Jiao Tong University School of Medicine (Shanghai, China). The C57BL/6-CD45.2 mice were ordered from LARC, Tongji University (Shanghai, China). All animal experiments were supported by our institution and conducted under the Guideline for Animal Care at Tongji University School of Medicine.

### Generation of the murine AML model

Bone marrow (BM) lineage-negative (Lin^−^) cells were sorted from mice and cultured overnight in 1640 medium supplemented with 20 % foetal bovine serum (FBS), 100 ng/mL stem cell factor (SCF), 10 ng/mL granulocyte colony-stimulating factor (G-CSF), 10 ng/mL interleukin-3 (IL-3), and 10 ng/mL interleukin-6 (IL-6). An MSCV-MLL-AF9-IRES-YFP-encoding plasmid and a PCL-ECO packaging plasmid were transfected into 293T cells to produce retroviruses. The above Lin^−^ BM cells were infected by two rounds of spin-occultation in the presence of 10 µg/ml polybrene. The infected bone marrow cells were transplanted into lethally irradiated (10.0 Gy) C57BL/6 mice by tail vein injection. Serial transplantations were performed with 2,000 sorted YFP^+^ c-Kit^+^ BM leukaemia cells from primary or secondary recipient mice and were transplanted into lethally irradiated (8.0 Gy) C57BL/6 mice by tail vein injection. Following transplantation, the recipients were provided acidified water (pH 1.3 to 2.0) for two weeks [23]. Where indicated, mice were administered tamoxifen (Sigma) in corn oil (20 mg/mL) daily by intraperitoneal injection for five consecutive days (150 mg per gram of body weight). For homing assays, lethally irradiated (10.0 Gy) recipients were transplanted with 10^6^ HCK^+/+^ or HCK^−/−^ BM leukaemia cells from the primary recipient mice, and the BM was analysed 16 h after transplant.

### Haematopoietic reconstruction and chimaerism assessment

Non-competitive transplants were performed with 10^6^ BM cells from HCK^+/+^ or HCK^−/−^mice, injected into lethally irradiated (10.0 Gy) B6-CD45.1 recipients. Chimaerism was assessed eight weeks after transplantation. To assess the chimaerism of mature cells in BM, antibodies against the following markers were used: Mac1-APC, Gr1- PeCy7, Ter119-APC, B220- PeCy7, CD3-APC, CD45.2-BV421, and CD45.1-BV421. To assess the chimaerism of stem cells and progenitor cells after transplantation, the following panel was used: lineage (CD3, CD4, CD8, Gr1, B220, CD19, and TER119)-APC, Sca1-PeCy7, CD34-FITC, c-KIT-PE, CD135-BV421, CD127-APC/BV421, CD16/32-BV421. All antibodies were purchased from BD Pharmigen and BioLegend. Stained cells were then analysed by the BD FACSVerse instrument, and the data were analysed by FlowJo 10. The following markers were used to define different cell populations: Long-term HSCs (LT-HSCs, Lin^−^C-kit^+^Sca-1^+^Flk2^−^CD34^−^), Short-term HSCs (ST-HSCs, Lin^−^C-kit^+^Sca-1^+^Flk2^−^CD34^+^), Multipotent progenitors (MPPs, Lin^−^C-kit^+^Sca-1^+^Flk2^+^CD34^+^), Common myeloid progenitors (CMPs, Lin^−^C-kit^+^Sca-1^−^CD16/32^low^CD34^+^IL7R^−^), Granulocyte-monocyte progenitors (GMPs, Lin^−^C-kit^+^Sca-1^−^CD16/32^+^CD34^+^IL7R^−^), Multiple myeloid progenitors (MEMEPs, Lin^−^C-kit^+^Sca-1^−^CD16/32^−^CD34^−^IL7R^−^), Common lymphoid progenitors (CLPs, Lin^−^C-kit^low^Sca-1^low^IL7R^+^), B cells (B220+), T cells (CD3^+^) and early erythroid cells (Ter119^+^). For homing assays, lethally irradiated (10.0 Gy) C57BL/6-CD45.1 recipients were transplanted with 10^6^ C57BL/6-CD45.2 HCK^+/+^ or HCK^−/−^ BM cells from the primary recipient mice, and the BM was analysed 16 h after transplant.

### Cell lines and cell culture

KG-1α, HEL, K562, NB4, THP-1, U937, MV4-11, and 293T cell lines were purchased from the Shanghai Cell Bank, Chinese Academy of Sciences (Shanghai, China). The 293T cell line was maintained in DMEM; the KG-1α, HEL, and K562 cell lines were cultured in IMDM; and the rest of the cell lines were cultured in RPMI-1640 medium. The mentioned media were supplemented with 10 % foetal bovine serum (FBS). The cells were cultured at 37 °C in a humidified atmosphere of 5 % CO_2_.

### **Lentivirus construction, infection, and phenotype analysis**

Lentiviral short hairpin RNAs (shRNAs) targeting human HCK, CDK6, and a scrambled shRNA (SCR) were constructed using a lentiviral vector, pLVX-shRNA2 (Clontech Laboratories, Inc., Mountainview, CA, USA). The interference sequences were as follows: shRNA-HCK-1, 5’- GCTGTGATTTGGAAGGGAA-3’; shRNA-HCK-2, 5’-GGATAGCGAGACCACTAAA-3’; shRNA-CDK6, 5’- GAGTAGTGCATCGCGATCTAA-3‘; shRNA-c-Myc-1, 5’- CAGTTGAAACACAAACTTGAA-3’; shRNA-Scramble, 5’-GTTCTCCGAACGTGTCACGT-3’. For the rescue experiments, the retroviral plasmid MSCV-IRES-mCherry was used. Lentiviruses were produced using the calcium phosphate transfection method with the packaging plasmids pCMV-dR8.91 dvpr (Δ89) and pCMV-VSV-G (VSVG). The lentiviral supernatant was used to infect several human leukaemia cell lines, which were subjected to proliferation, colony formation and signalling pathway analyses in vitro at the indicated time points.

### Colony-forming assays

A colony assay for murine LSCs was performed by plating 500 sorted YFP^+^c-Kit^+^ LSCs on methylcellulose (MethoCult M3434, Stem Cell Technologies), and colonies were counted 14 days later. A standard two-layer soft agar culture was used for the colony-forming assay of the human cell lines (0.6 % agarose bottom layer and a 0.3 % agarose top layer). The cells were seeded at 10^5^/ml in 24-well plates with soft agar. Similarly, colonies were imaged and counted 14 days after plating.

### Western blotting

Cell lysates of fluorescence-activated cell sorting (FACS)-purified HCK^+/+^ or HCK^−/−^ YFP^+^ c-Kit^+^ LSCs, shRNAs targeting human HCK and CDK6, CDK6-overexpressing THP-1 cells, and their control cells were electrophoresed on 10 % SDS polyacrylamide gels and transferred onto PVDF membranes (Millipore). The membranes were blocked with 5 % non-fat milk and reacted with the indicated primary antibodies, followed by incubation with appropriate HRP-conjugated secondary antibodies. The primary antibodies were as follows: anti-HCK (Abcam, ab124245), anti-CDK6 (Cell Signaling Technology, 3136), anti-c-Myc (Cell Signaling Technology, 18,583), anti-MAPK1/2 (Cell Signaling Technology, 4695), anti-p-MAPK1/2 (Cell Signaling Technology, 4376), and anti-β-Actin (Cell Signaling Technology, 3700).

### RT-PCR

Total RNA was extracted from cells using a Quick-RNA™ Microprep kit (Zymo). An equal amount of RNA from the samples was reverse transcribed into cDNA with a FastQuant RT Kit (TIANGEN), and qPCR was performed using an ABI 7500 sequence detection system using the primers listed in Supplemental Table 2. All primers were obtained from PrimerBank [24].

### Proliferation assay for human cell lines

Cells were plated at 10,000 cells/ml in a 24-well plate for the proliferation assay. Cells were counted for six consecutive days.

### Cell Counting Kit-8 (CCK-8) assay of human cell lines

Cells were seeded in 96-well plates for cell viability analysis using Cell Counting Kit-8 (CCK-8; Dojindo). CCK-8 assays were performed with three replicates, and the optical density (OD) values at 450 nm were measured using a microplate imaging system.

### Apoptosis analysis

Apoptosis was analysed by staining with Annexin V and propidium iodide (BD Biosciences) according to the manufacturer’s instructions. Flow cytometry analysis was then performed within 1 h. The cell apoptotic ratio was detected by a FACSVerse cytometer (BD Biosciences) and was analysed by FlowJo 10.

### Cell cycle analysis in vivo and in vitro

We used immunofluorescent staining of incorporated BrdU (BD Biosciences) and flow cytometric analysis to determine the frequency and nature of individual cells that have synthesized DNA. In vivo, intraperitoneal injection of BrdU (2 mg at 10 mg/mL) for 4 h. In vitro, the addition of BrdU (5 µg/ml) for 2 h.

### Statistical analysis

All quantitative data are represented as the mean ± SEM. Unpaired 2-tailed Student’s t-test was used to assess differences between two independent groups. The Mann-Whitney U test was used to assess differences between nonparametric data, and one-way analysis of variance (ANOVA) was used to assess multigroup comparisons. For the Kaplan-Meier survival analysis, the log-rank test was used. Differences with a *p*-value less than 0.05 were considered statistically significant (*P < 0.05; **P < 0.01; ***P < 0.001). The data were analysed using GraphPad Prism 8.

## Results

### 1. HCK is highly enriched in LSCs and correlates with survival

**Fig. 1:**
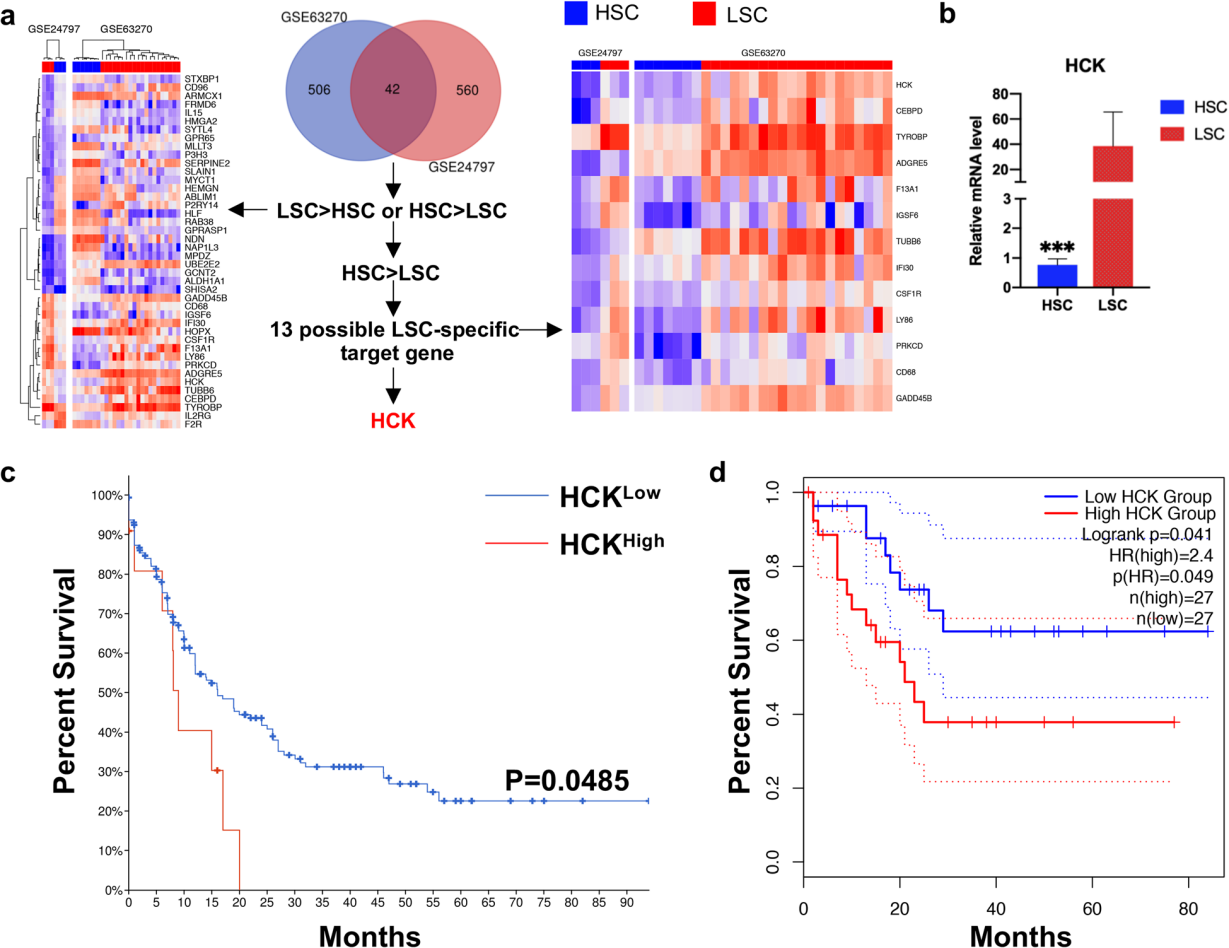
HCK is highly enriched in LSCs, and correlates with poor survival.** (a)** The analysis for integrating expression profiles obtained from two independent array platforms (GSE63270 and GSE24797). The heat maps represent microarray signal intensity on a log2 scale. **(b)** Comparison of the expression level of HCK in CD34^+^CD38^−^ cells. **(c-d)** Overall survival of AML patients stratified into HCK^Low^ and HCK^High^ cohorts

Results of the CCLE analysis showed the level of HCK mRNA transcripts in AML ranks 1th among many cancer cell lines (Supplemental Fig. 2a). Meanwhile, we also found that the level of HCK mRNA expression in AML tissues was higher than in normal tissues through GEPIA dataset analysis (Supplemental Fig. 2b). Ideally, a therapeutic strategy that targets LSCs must spare HSCs to protect normal haematopoiesis in AML patients. Therefore, the comparison of LSCs and HSCs is crucial to identify target molecules specific to LSCs. Researchers previously identified HCK, which is highly enriched in dormant LSCs taken from the BM of AML patients[19]. We performed gene expression analyses with two independent array platforms: GSE24797[25] and GSE63270[26]. The strategies used to integrate the gene expression data obtained with the two platforms are summarized in Fig. 1a. The rank product method was performed to extract genes with expression levels that were significantly higher in LSCs than in HSCs. We reached the same conclusion: HCK is highly enriched in LSCs compared with HSCs (Fig. 1a). To verify this finding, we sorted CD34^+^CD38^−^ cells from BM of people with AML (denoted as LSCs), and sorted CD34^+^CD38^−^ from umbilical cord blood (denoted as HSCs). The results showed that the expression of HCK in AML CD34^+^CD38^−^ cells was much higher than CD34^+^CD38^−^ cells in umbilical cord blood, which confirmed that HCK is highly enriched in LSCs (Fig. 1b).

Furthermore, we categorized human AML samples into HCK^High^ and HCK^Low^ subgroups by applying the cBioPortal database. Surprisingly, there was a strong correlation between HCK gene expression and poor patient prognosis (Fig. 1c). The median overall survival of the HCK^High^ cohort was 8.97 months, and that of the HCK^Low^ cohort was 15.93 months. We also reached similar conclusions via GEPIA dataset analysis (Fig. 1d).

### 2. HCK is required for the proliferation of human AML cell lines

**Fig. 2:**
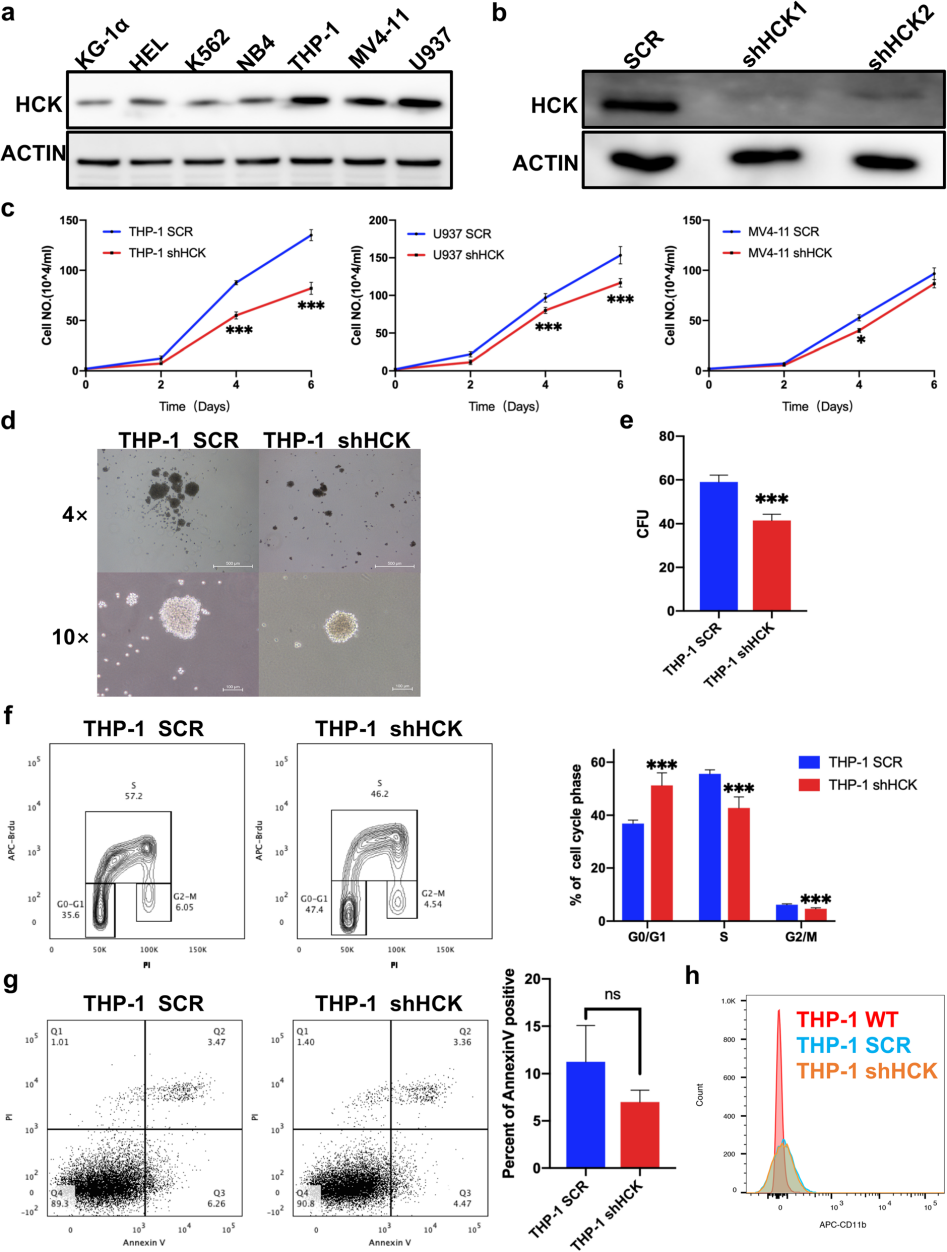
HCK is required for the proliferation of human AML cell lines.** (a)** Immunoblotting analysis of HCK expression in different leukaemia cell lines, including KG-1α, HEL, K562, NB4, THP-1, U937, and MV4-11. **(b)** Cells were transfected with shRNAs targeting HCK, followed by immunoblotting for HCK. **(c)** The numbers of THP-1, U937, and MV4-11 cells were counted at the indicated days after infection with shRNA targeting HCK or SCR. **(d)** Representative images of colonies formed by HCK-knockdown THP-1 cells after 14 days. **(e)** Quantification of colony numbers in d. **(f)** Representative flow cytometric analysis of the cell cycle distribution in THP-1 cells transfected with shRNA targeting HCK or SCR was determined using a BrdU incorporation assay. **(g)**Representative flow cytometric analysis of apoptosis in THP-1 cells transfected with shRNA targeting HCK or SCR. **(h)**Representative flow cytometric analysis of the differentiation in THP-1 cells transfected with shRNA targeting HCK or SCR

To further evaluate the functions of HCK in human AML, we first measured the protein levels of HCK in different types of human AML cell lines by western blot analysis. As shown in Fig. 2a, HCK was highly enriched in U937, THP-1, and MV4-11 cells. We further decided to construct shRNAs to knock down HCK for testing its roles in these cell lines. As shown in Fig. 2b, shHCK1 and shHCK2 efficiently downregulated the protein levels of HCK in THP-1 cells, especially shHCK1, as evaluated by western blot. The cells infected with shHCK expanded more slowly than those infected with SCR, as shown in Fig. 2c. A functional assay showed that HCK-knockdown THP-1 cells gave rise to fewer colonies in vitro (Fig. 2d, e). Mechanistically, HCK-knockdown THP-1 cells tended to arrest in the G1 phase, as determined by bromodeoxyuridine (BrdU) incorporation analysis (Fig. 2f). Interestingly, HCK-knockdown THP-1 cells did not lead to an increase in apoptosis and did not lead to differentiation (Fig. 2 g, h). Similar results were also found in U937 and MV4-11 cells (Supplemental Fig. 3a-f).

AML was a biologically and clinically heterogeneous disease. The levels of HCK in various AML cell lines were not identical. The therapeutic efficacy of SFK inhibitors will be influenced by the extent of SFK activity in tumors [27]. To clarify the role of HCK in other AML cell lines with low HCK expression, we knocked down HCK in KG-1a cell lines (Supplemental Fig. 4a). As shown in Supplemental Fig. 4b-c, the absence of HCK did not affect the proliferation and cell cycle progression of KG-1a.

### HCK is required for leukemogenesis and self-renewal abilities of LSCs

**Fig. 3:**
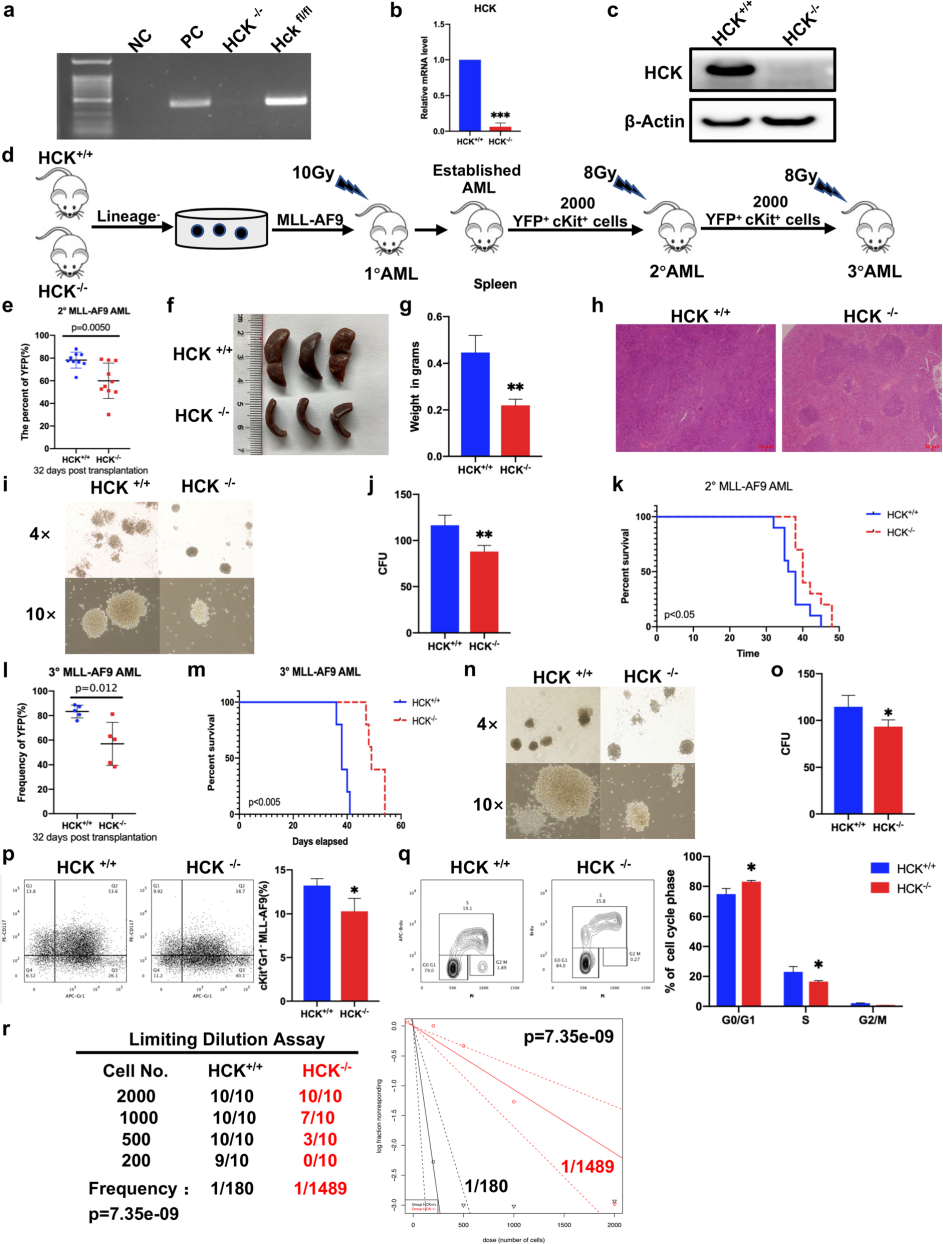
HCK is required for leukaemogenesis and self-renewal of LSCs.** (a-c)** Genome PCR, RT-PCR, and western blotting were performed with HCK ^−/−^ BM cells to confirm HCK deletion. NC: Negative control; PC: Positive control.  **(d)** Experimental scheme for investigating the function of HCK in leukaemogenesis. **(e)** Frequency of YFP^+^ leukaemia cells in the peripheral blood at 32 days after the second transplantation. **(f)** Representative images of the sizes of spleens of recipient mice upon the second transplantation. **(g)** Quantification of the weights of the spleens in f. **(h)** Histological H&E staining of the spleens in f. **(i)** Representative images of colonies formed by YFP^+^c-Kit^+^ LSCs from secondary recipients. **(j)** The colony numbers were calculated in i. **(k)** Survival data for recipient mice receiving HCK^−/−^ or HCK^+/+^ YFP^+^c-Kit^+^ LSCs upon the second transplantation (*n *= 10; log-rank test). **(l)** Frequency of YFP^+^ leukaemia cells in the peripheral blood at 32 days after the third transplantation. **(m)** Survival data for recipient mice receiving HCK^−/−^ and HCK^+/+^ YFP^+^c-Kit^+^ LSCs upon the third transplantation (*n* = 5; log-rank test). **(n)** Representative images of colonies formed by YFP^+^c-Kit^+^ LSCs from the third transplant recipient. **(o)** The colony numbers were calculated in n. **(p)** Representative FACS plot and graph of c-Kit and Gr1 expression in HCK^−/−^ and HCK^+/+^ leukaemic cells to assess the frequency of LSC. **(q)** Cell cycle status was determined in the third transplant recipient in vivo. **(r)** LSC frequency was determined from a limiting dilution assay performed with BM cells from the third transplant recipient mice. The ELDA web tool was used to calculate the frequency of LSCs

To test the function of HCK in vivo, we used mice with a complete deletion of HCK, which were called “HCK-knockout first” mice (denoted as HCK^−/−^) (Supplemental Fig. 1a). We assessed the importance of HCK in leukaemogenesis by using a mixed-lineage leukaemia fusion protein (MLL-AF9)-driven mouse model of AML. HCK deletion was confirmed by genomic PCR, reverse transcription-PCR (RT-PCR), and western blotting (Fig. 3a-c). Lineage-negative cells were transduced with the MLL-AF9 retrovirus from HCK^+/+^ and HCK^−/−^ mice to generate an MLL-AF9-driven leukaemia model (Fig. 3d, Supplemental Fig. 5a-c). In the first transplant, there was no significant difference between HCK^+/+^ and HCK^−/−^ AML mice (Supplemental Fig. 5d, e). We analysed the homing ability of HCK^−/−^ leukaemia cells. We found no significant difference in the frequencies of HCK^+/+^ and HCK^−/−^ that homed to the BM 16 h after injection (Supplemental Fig. 5f). LSCs were marked in this model as YFP^+^ c-Kit^+^ or YFP^+^ c-Kit^+^ Gr1^−^ [28–31]. Therefore, the second transplantation was performed with 2,000 sorted YFP^+^c-Kit^+^ BM leukaemia cells of primary recipient mice and 10^^^6 support cells (Fig. 3d). The frequency of YFP^+^ leukaemia cells in the peripheral blood of the HCK^−/−^ group was lower than that in peripheral blood of the HCK^+/+^ group at 32 days after the second transplantation (Fig. 3e), which was consistent with a decrease in the sizes and weights of the spleens of HCK^−/−^ leukaemic recipient mice (Fig. 3f, g, h). HCK^−/−^ mice exhibited a significant decrease in the number of YFP^+^c-Kit^+^ LSC colonies formed in vitro (Fig. 3i, j). More importantly, the median survival of the HCK^−/−^ group was significantly extended compared to that of the HCK^+/+^ group (40 vs. 37 days) (Fig. 3k), indicating that HCK played an important role for AML leukemogenesis.

To further assess the self-renewal function of HCK in LSCs, we performed third transplantation with the same number of LSCs. As shown in Supplemental Fig. 5 g, the levels of HCK in mouse LSC in the 2nd and 3rd transplantations were extremely low. The frequency of YFP^+^ leukaemia cells in the peripheral blood of the HCK^−/−^ group was lower than that in the peripheral blood of the HCK^+/+^ group at 32 days after the second transplantation (Fig. 3 L). Consistently, recipient mice receiving HCK^−/−^ LSCs had remarkably delayed survival times during the third transplantation (49 vs. 38 days of the median survival) (Fig. 3 m). Furthermore, HCK^−/−^ mice also exhibited a significant decrease in the number of YFP^+^ c-Kit^+^ LSC colonies formed in vitro (Fig. 3n, o), and the frequency of LSCs (YFP^+^ c-Kit^+^ Gr1^−^) in the BM also decreased (Fig. 3p). Moreover, there was a markedly increased proportion of HCK^−/−^ LSCs in the G1 phase and a decrease in HCK^−/−^ LSCs in the S phase (Fig. 3q, Supplemental Fig. 5 h).

As we observed that the self-renewal ability was dramatically reduced upon the third transplantation, we determined the LSC frequencies in the HCK^+/+^ and HCK^−/−^ leukaemia cells of third recipient mice by a limiting dilution analysis. We found that the deletion of HCK resulted in a decrease in the frequency of LSCs compared with the WT counterparts (1/1489 vs. 1/180) (Fig. 3r).

### 4. HCK is required for AML maintenance

**Fig. 4:**
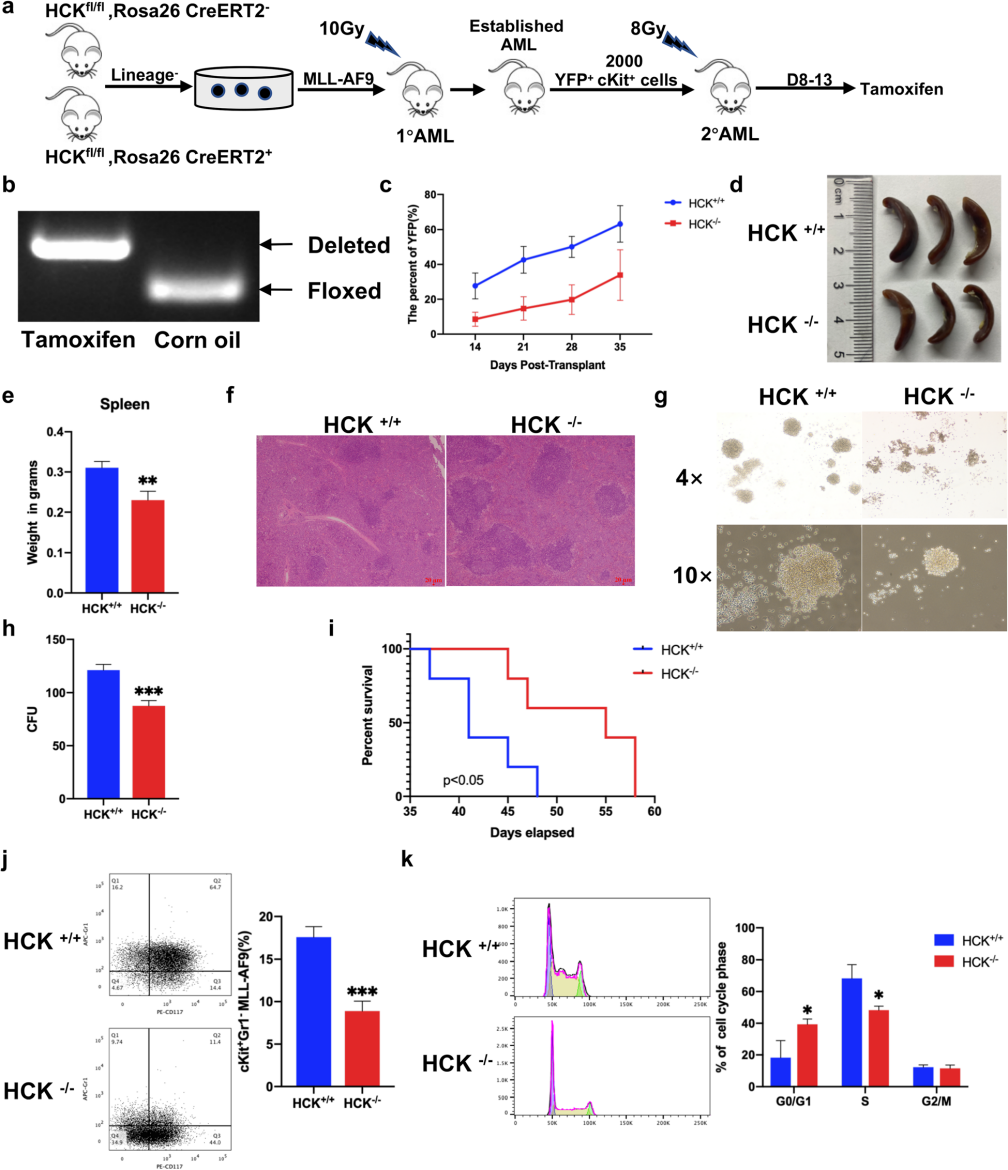
HCK is required for AML maintenance.** (a)** Experimental scheme for investigating the function of HCK in AML maintenance. **(b)** PCR-based confirmation of HCK deletion in MLL-AF9 leukaemia cells following tamoxifen delivery in vivo. **(c)** Frequency of YFP^+^ leukaemia cells in the peripheral blood at the indicated time after the second transplantation (n = 10). **(d)** Representative images of the sizes of spleens of recipient mice. **(e)** Quantification of the weights of the spleens in d. **(f)** Histological H&E staining of the spleens in d. **(g-h)** Representative images and number of colonies formed by YFP^+^c-Kit^+^ LSCs from the secondary recipients. **(i)** Survival data upon the secondary recipient mice (n = 10; log-rank test). **(j)** Representative FACS plot and graph of c-Kit and Gr1 expression in HCK^−/−^ and HCK^+/+^ MLL-AF9 leukaemic cells to assess the frequency of LSCs. **(k)** Representative flow cytometric analysis of the cell cycle distribution in HCK^−/−^ and HCK^+/+^ leukaemic cells from the second transplant recipient in vitro

To determine whether continued AML maintenance requires HCK, we crossed HCK^−/−^ mice with FLP (H11-CAG-FLPO) mice and Rosa26-CreERT2 mice successively to generate HCK^fl/fl^ /Rosa26-CreERT2 mice (Supplemental Fig. 1a, b). Lineage-negative cells from HCK^fl/fl^ /Rosa26-CreERT2 or HCK^fl/fl^ Rosa26 mice were retrovirally transduced with MLL-AF9-IRES-YFP and transplanted to establish AML. Second transplantation was performed with 2,000 sorted YFP^+^c-Kit^+^ BM leukaemia cells of the primary recipient mice and 1* 10^^^6 support cells, recipients treated with tamoxifen from day 8 to day 13 (Fig. 4a). Cre-mediated recombination leads to the excision of exon 3, which results in complete loss of HCK expression (Fig. 4b). The frequency of YFP^+^ leukaemia cells in the peripheral blood of the tamoxifen-induced HCK^−/−^ group was lower than that in the peripheral blood of the HCK^+/+^ group (Fig. 4c), which was consistent with a decrease in the sizes and weights of the spleens of HCK^−/−^ leukaemic recipient mice (Fig. 4d, e, f). Tamoxifen-induced HCK loss led to a reduction in the number of leukaemic colonies formed in vitro (Fig. 4 g, h) and significantly increased the median survival of mice (55 vs. 41 days) (Fig. 4i). The frequency of LSCs (YFP^+^ c-Kit^+^ Gr1^−^) in the BM also decreased (Fig. 4j). Furthermore, there was also an increased proportion of tamoxifen-induced HCK^−/−^ LSCs in the G1 phase and a decrease in the proportion of HCK^−/−^ LSCs in the S phase (Fig. 4k). Taken together, these data demonstrate that HCK contributes to AML maintenance in murine models of MLL-AF9-driven AML.

### 5. HCK maintains the CDK6 level to promote cell proliferation

**Fig. 5:**
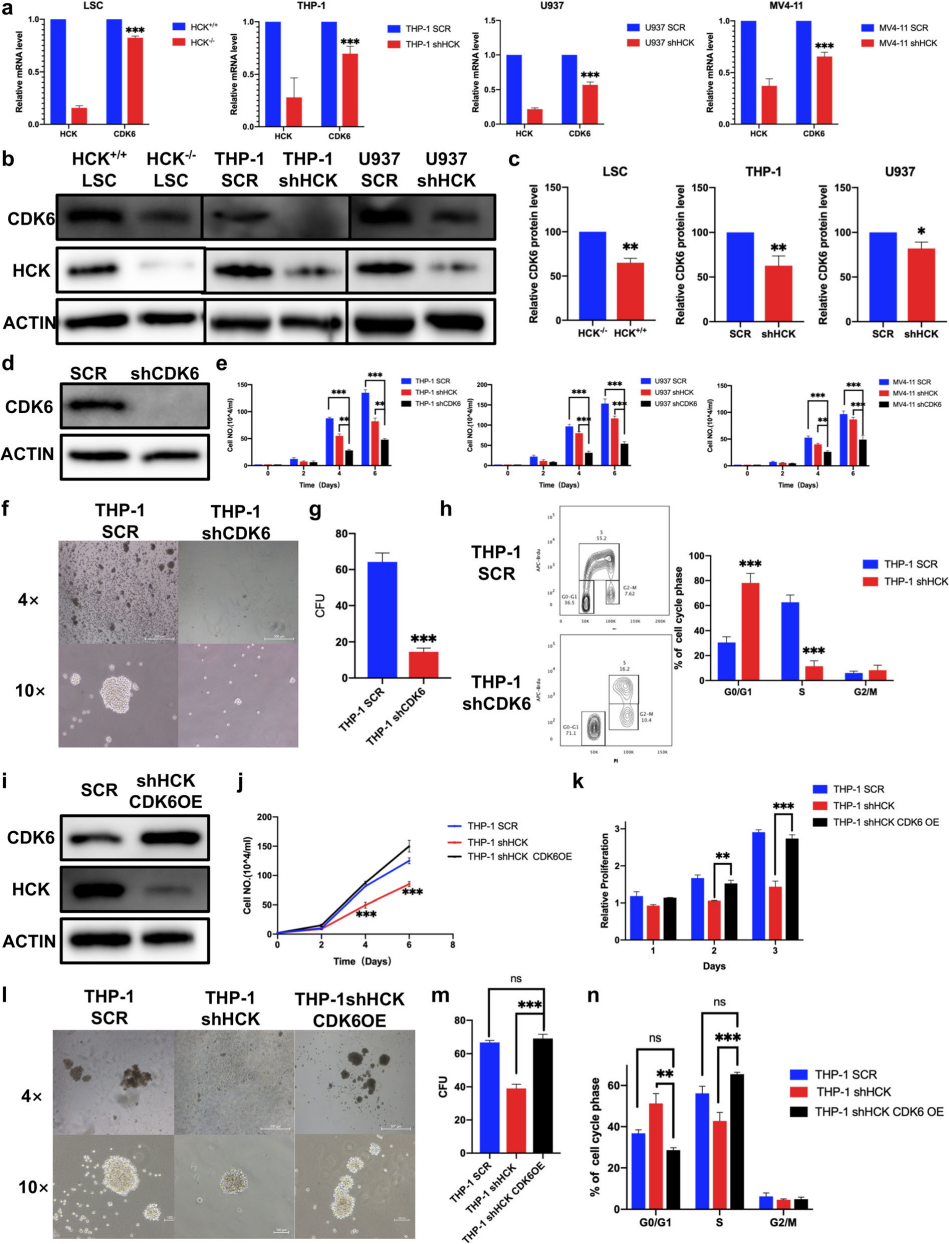
HCK maintains the CDK6 level to promote cell proliferation.** (a)** CDK6 expression decreased in both human cell lines and BM LSCs after HCK deficiency, as determined by RT-PCR. **(b)** Immunoblotting analysis of CDK6 expression after HCK deficiency in LSCs, THP-1 and U937. **(c)** Quantification of CDK6 protein level by densitometric analysis. **(d)** shRNA targeting CDK6, followed by immunoblotting for CDK6. **(e)** The numbers of cells were counted at the indicated days after infection with shRNA targeting CDK6, HCK or scrambled shRNA. **(f)** Representative images of colonies formed by CDK6-knockdown THP-1 cells after 14 days. **(g)** The colony numbers were calculated in g. **(h)** Representative flow cytometric analysis of the cell cycle distribution in THP-1 cells targeted by shHCK or SCR was determined using a BrdU incorporation assay. **(i)** HCK-knockdown and CDK6 overexpression, followed by immunoblotting for HCK and CDK6. **(j)** The numbers of THP-1 cells were counted at the indicated days after infection with shRNA targeting HCK, with shRNA targeting HCK and plasmids encoding CDK6, or with scrambled shRNA. **(k)** The relative proliferation of THP-1 cells on the indicated days after infection with shRNA targeting HCK, with shRNA targeting HCK and plasmids encoding CDK6, or with scrambled shRNA by Cell Counting Kit-8 assays. **(l)** Representative images of colonies formed by THP-1 cells after infection with shRNA targeting HCK, with shRNA targeting HCK and plasmids encoding CDK6, or with scrambled shRNA. **(m)** The colony numbers were calculated in m. **(n)** The cell cycle distribution of THP-1 cells infected with shRNA targeting HCK, with shRNA targeting HCK and plasmids encoding CDK6, or with scrambled shRNA

In this study, HCK-knockdown cells, including human cell lines and LSCs, tended to arrest in the G1 phase. To unravel the underlying molecular mechanisms that control self-renewal and cell cycle arrest in HCK^−/−^ LSCs, we applied RT-PCR to search the potential candidates related to self-renewal and cell cycle (Supplemental Fig. 6a). Moreover, we also detected changes in the expression of SFKs in LSCs after the deletion of HCK (Supplemental Fig. 6b). We found that CDK6 expression decreased in both human cell lines and BM LSCs after HCK knockdown or deletion (Fig. 5a-c). To confirm the dependence of AML cells on CDK6, we constructed shRNA to knock down CDK6 (Fig. 5d). We found that CDK6-knockdown significantly inhibited cell proliferation compared to HCK-knockdown (Fig. 5e). In addition, CDK6-knockdown inhibited the colony formation (Fig. 5f, g). Naturally, the cell cycle was also arrested in G1 (Fig. 5 h). To determine whether CDK6 was the critical regulator gene of HCK function, we transferred MSCV-IRES-huCDK6-mCherry plasmid in HCK-knockdown THP-1 cells (Fig. 5i). We found that cell proliferation (Fig. 5j, k) and colony formation (Fig. 5 L, m) was restored and that cell cycle arrest was attenuated (Fig. 5n) compared with HCK-knockdown THP-1 cells. Combining all the results, we can conclude that HCK maintains the CDK6 level to promote proliferation and self-renewal.

### HCK may regulate CDK6 expression through the ERK1/2-c-Myc axis

**Fig. 6:**
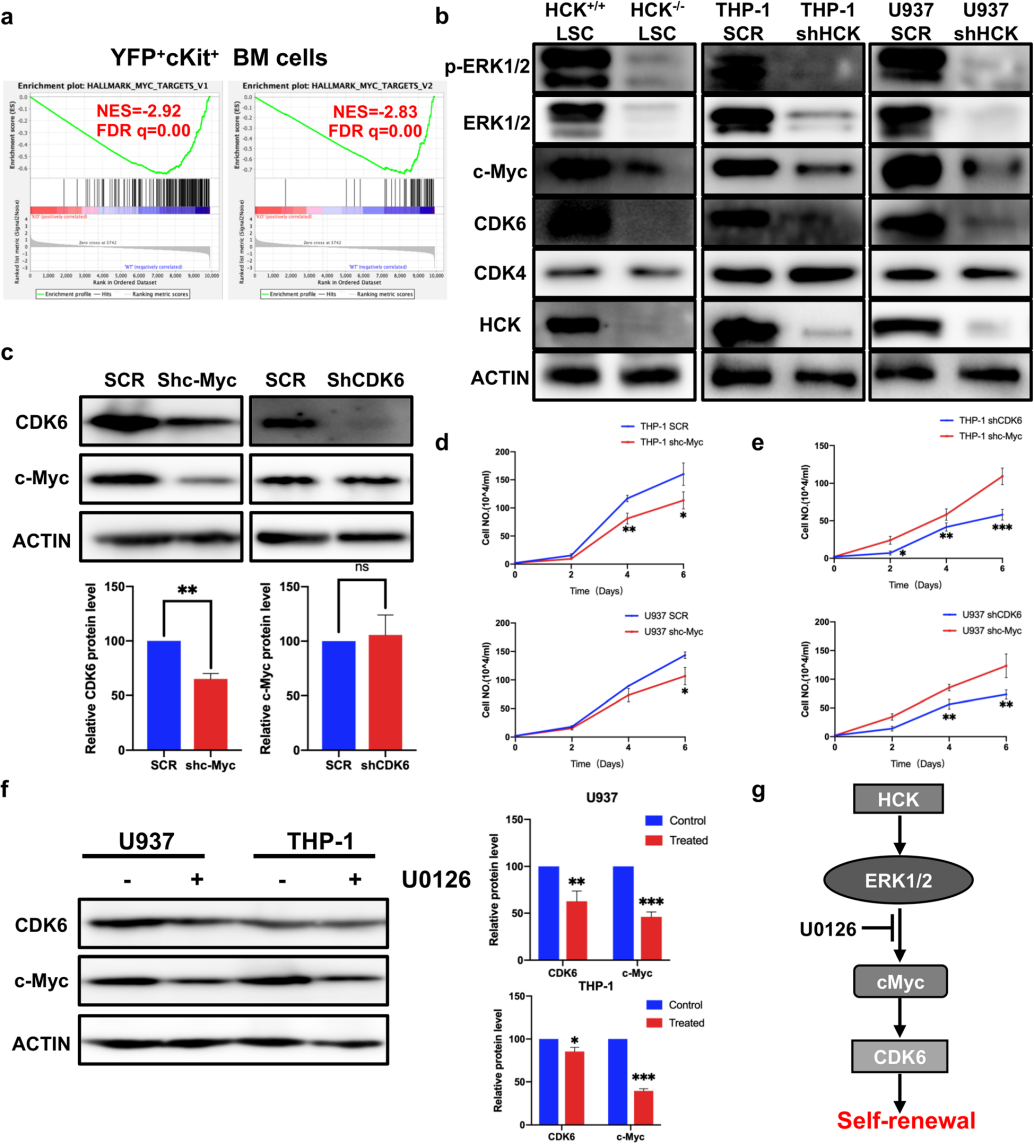
HCK may regulate CDK6 expression through the ERK1/2-c-Myc axis.** (a)** GSEA plots evaluating changes in c-Myc dependent gene signatures upon HCK deletion. FDR q-val, false discovery rate q-value; NES, normalized enrichment score. **(b)** Immunoblotting for HCK, CDK4, CDK6, c-Myc, ERK1/2, and p-ERK1/2 in LSCs, THP-1 and U937 cells. **(c)** Transfection of cells with shRNAs targeting c-Myc or CDK6, followed by immunoblotting for c-Myc and CDK6 and quantification of c-Myc or CDK6 protein level by densitometric analysis. **(d)** The numbers of THP-1 and U937 cells were counted on the indicated days after infection with shRNA targeting c-Myc or with scrambled shRNA. **(e)** The numbers of THP-1 and U937 cells were counted on the indicated days after infection with shRNA targeting c-Myc or with shRNA targeting CDK6. **(f)** Immunoblotting for CDK6 and c-Myc after treatment with an ERK1/2 inhibitor in U937 or THP-1, and quantification of c-Myc or CDK6 protein level by densitometric analysis. **(g)** Schematic representation of the HCK—ERK1/2—c-Myc—CDK6 protein network in AML

In our study, HCK affected the cell cycle by regulating the expression of CDK6, thereby affecting the self-renewal of LSCs. To better research the function of HCK in self-renewal, we performed RNA sequencing of leukaemia stem cells taken from the third recipient mice. Through GSEA plot evaluation, we found the systematic suppression of c-Myc target gene expression upon HCK deletion (Fig. 6a). We performed protein analysis upon LSCs and human cell lines through western blotting. We found that after HCK loss, the expression of both CDK6 and c-Myc was reduced to a certain extent (Fig. 6b). To better understand the relationship between c-Myc and CDK6, we constructed shRNA to knock down c-Myc. We found when the expression of c-Myc decreased, the expression of CDK6 also reduced, while when the expression of CDK6 decreased, the change of c-Myc was not apparent (Fig. 6c). The proliferation ability of THP-1 cells was reduced after c-Myc-knockdown (Fig. 6d). Moreover, the proliferation ability of CDK6-knockdown THP-1 cells was reduced compared with c-Myc-knockdown THP-1 cells (Fig. 6e). Therefore, we considered that c-Myc might be an upstream regulatory gene of CDK6 and regulated by HCK. To understand how HCK affects the c-Myc/CDK6 pathway, we also examined the level of a critical upstream regulator, ERK1/2, by immunoblotting. The results showed that both the phospho-ERK1/2 and total ERK1/2 protein levels were markedly reduced in HCK^−/−^ LSCs or human AML cell lines compared to controls (Fig. 6b). To further determine whether ERK signalling can regulate c-Myc and CDK6 and affect the phenotypes of LSCs and human AML cell lines, we used ERK1/2 inhibitors (U0126, 20 µM) in THP-1 cells. We found that both c-Myc and CDK6 were downregulated to a certain extent (Fig. 6f). Therefore, we considered that HCK might regulate CDK6 expression through the ERK1/2-c-Myc axis (Fig. 6 g).

### 7. HCK is dispensable for normal haematopoiesis

**Fig. 7:**
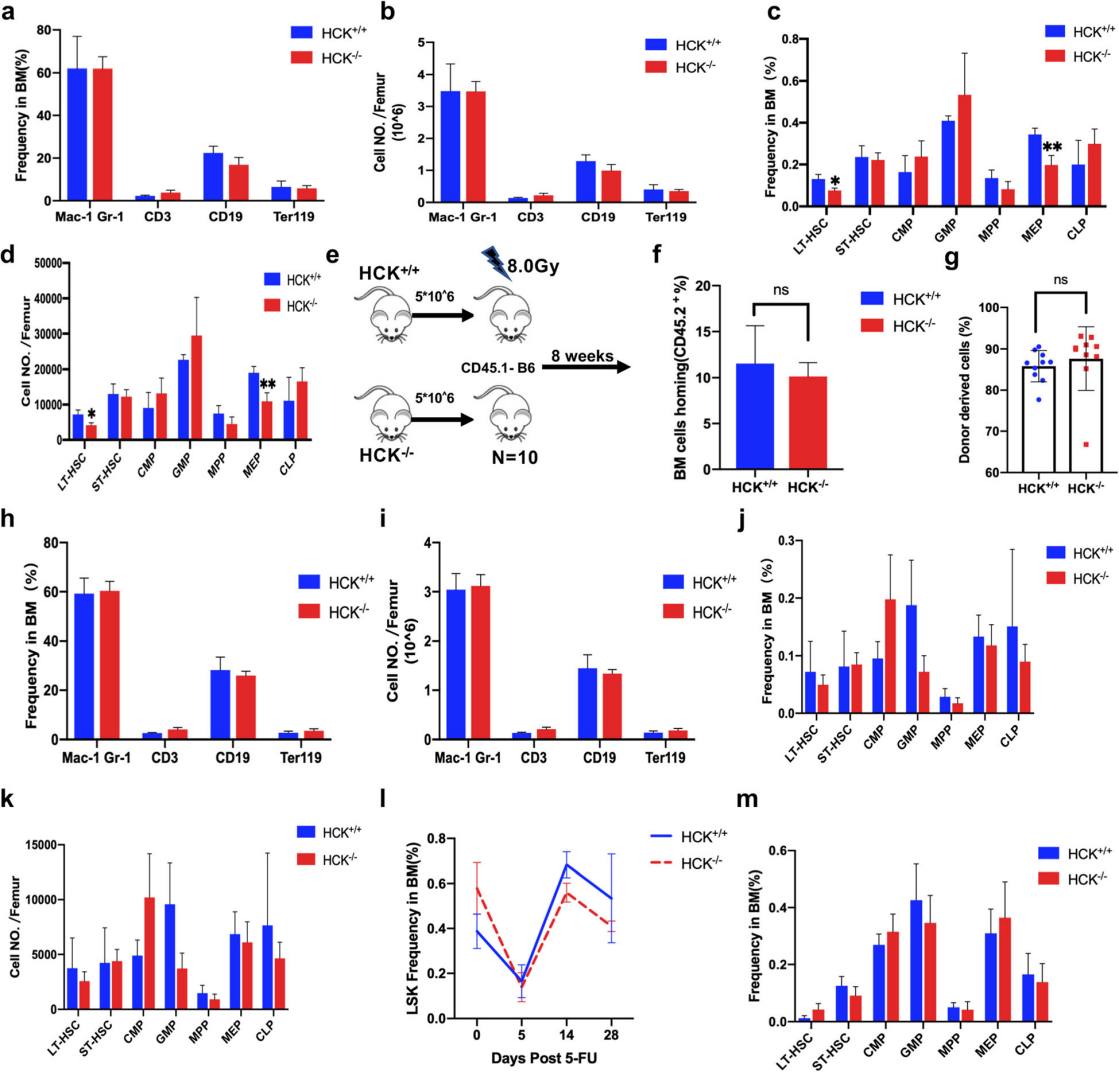
HCK deletion has almost no effect on normal haematopoiesis.** (a-b)** Average frequencies and numbers of differentiated cells in HCK^−/−^ and HCK^+/+^ mice. **(c-d)** Average stem cell frequencies and absolute numbers of stem and progenitor cells in HCK^−/−^ and HCK^+/+^ mice. **(e)** Experimental scheme for investigating normal haematopoietic reconstruction. **(f)** No defect was found in the homing of HCK^−/−^ BM cells to the BM. **(g)** Chimaerism assessment in peripheral blood (n = 10). **(h-i)** Average frequencies and numbers of differentiated cells in the reconstruction of the haematopoietic cell system in HCK^−/−^ and HCK^+/+^ mice. **(j-k)** Average stem cell and progenitor cell frequencies and absolute numbers of stem cells and progenitor cells in the reconstruction of the haematopoietic cell system in HCK^−/−^ and HCK^+/+^ mice. **(l)** Frequency of LSK cells in bone marrow on the indicated days after 5-Fu injection. **(m)** Average stem cell and progenitor cell frequencies in in HCK^−/−^ and HCK^+/+^ mice at day 28 after 5-Fu injection

In our study, HCK played a critical role in maintaining the self-renewal ability of LSCs by regulating the expression of CDK6. HCK may be a promising target for treatment. Ideally, a therapeutic strategy that targets LSCs must spare HSCs to protect normal haematopoiesis. Therefore, whether HCK affected normal haematopoiesis was our next research topic. HCK-knockout mice displayed a relatively mild phenotype, but little is known about its role in normal haematopoiesis [32]. To examine the role of HCK in HSCs, we assessed different populations of haematopoietic cells in HCK ^−/−^ and HCK wild-type (denoted as HCK^+/+^) littermates. There was no significant difference in the frequency or the absolute number of myeloid cells, B cells, T cells, or early erythroid progenitors between HCK ^−/−^ and HCK^+/+^ BM (Fig. 7a, b). HCK deletion did not adversely affect short-term HSCs (ST-HSCs) (Lin^−^C-kit^+^Sca-1^+^Flk2^−^CD34^+^), multipotent progenitors (MPPs) (Lin^−^C-kit^+^Sca-1^+^Flk2^+^CD34^+^), common myeloid progenitors (CMPs) (Lin^−^C-kit^+^Sca-1^−^CD16/32^low^CD34^+^IL7R^−^), granulocyte-monocyte progenitors (GMPs) (Lin^−^C-kit^+^Sca-1^−^CD16/32^+^CD34^+^IL7R^−^), or common lymphoid progenitors (CLPs) (Lin^−^C-kit^low^Sca-1^low^IL7R^+^) (Fig. 7c, d). Some changes, such as a reduction in long-term HSCs (LT-HSCs) (Lin^−^C-kit^+^Sca-1^+^Flk2^−^CD34^−^) and megakaryocyte-erythrocyte progenitors (MEPs) (Lin^−^C-kit^+^Sca-1^−^CD 16/32^−^CD34^−^IL7R^−^), were noted and could be due to alterations in differentiation or proliferation (Fig. 7c, d).

Non-competitive transplantation was applied to study the effect of HCK deletion on normal haematopoietic reconstruction. BM cells from HCK^−/−^ and HCK^+/+^ mice were transplanted into irradiated (8.0 Gy) CD45.1 congenic recipient (Fig. 7e). In our study, HCK deletion did not affect haematopoietic cell homing to BM (Fig. 7f). Deletion following stable reconstitution two months after transplant resulted in equivalent chimaerism in the peripheral blood (Fig. 7 g). Furthermore, there were no significant differences in the frequency and the absolute number of mature cells (myeloid cells, B cells, T cells, and early erythroid progenitors), HSCs, and haematopoietic progenitors (Fig. 7 h, i, j, k). From these results, we can conclude that HCK is dispensable for normal haematopoiesis.

In order to explore the regulatory effect of HCK on hematopoietic stem cells under stress state, HCK+/+ and HCK-/- mice were intraperitoneally injected with 5-Fu at a dose of 100 mg/kg, and antibody was labelled on the cell surface at d5, d14, and d28 after injection, respectively. The number of LSK cells in bone marrow was analysed by flow cytometry. As shown in Fig. 7 L, after 5 days of 5-Fu treatment, hematopoietic stem cells in bone marrow decreased sharply, and proliferation reached A peak two weeks after injection. Then, the hematopoietic function gradually returned to normal. The results showed that the loss of HCK did not affect the proliferation of hematopoietic stem cells after the hematopoietic system was damaged by 5-Fu. In addition, the absence of HCK did not affect the recovery of the hematopoietic system, as shown in Fig. 7 m.

## Discussion

AML is the first haematopoietic malignancy shown to depend on LSCs, which are responsible for drug resistance and relapse [8, 12]. Standard chemotherapy rarely triggers durable remission in AML, and targeting LSCs is a promising strategy for clinical AML therapy [12, 33]. In this study, we revealed that HCK positively regulates LSC self-renewal in part through CDK6, a cyclin-dependent kinase. Functional roles of HCK in the self- renewal ability of LSCs, leukemogenesis and leukaemia maintenance have not previously been reported.

HCK is a member of the Src family of tyrosine kinases, just expressed in cells of the myeloid and B-lymphoid lineages [13]. HCK helps couple the Fc receptor to activate the respiratory burst and plays a role in neutrophil migration and the degranulation of neutrophils [16, 34]. Although its physiological significance is clear, the functional roles of HCK in AML cells, especially in LSCs, have not been elucidated. But here, we observed that HCK modulated LSC self-renewal by regulating CDK6, suggesting that both HCK and CDK6 are potential therapeutic targets of LSCs.

To understand the mechanistic link that connects the HCK expression to the self-renewal program, we focused on CDK6. Extensive studies implicating the importance of CDK6 in AML [35–37]. Previous studies had reported an pathway linked FLT3-ITD, HCK and CDK6 in AML [38]. However, The functional relationship between HCK and CDK6 is unclear yet. We found that HCK depletion results in reduced expression of CDK6, which in turn leads to cycle arrest. Furthermore, the phenotypes resulting from HCK deficiency can be rescued by CDK6 overexpression, supporting that HCK maintains the self-renewal of leukaemia stem cells via CDK6 in AML. Together with recently published research on MLL-rearranged AML, our study suggests that CDK6 plays a central role in AML [39]. AML cells show increased activation of the CDK6, suggesting that the deregulation of the CDK6 is a hallmark of AML. In addition to CDK6, cell cycle regulators, such as CCND1 or CCND2, also differentially affect the maintenance of LSC self-renewal activity [35, 37, 40]. Whether other cell cycle regulators are also involved in LSC self-renewal requires further investigation.

ERK1/2 responds to a variety of internal and external stimuli and plays a crucial role in cell cycle progression, proliferation, chemical resistance, cell apoptosis, migration, and invasion [41]. c-Myc is the most essential member of the MYC oncogene family and is involved in a variety of biological functions of cancer, including proliferation, metastasis, apoptosis, differentiation, and metabolism [42]. As a transcription factor, c-Myc is constitutively overexpressed in various types of cancer, leading to the transcription of specific target genes, leading to the malignant progression of cancer [43]. Although c-MYC is a promising target for drug development, the direct targeting of c-Myc has been hindered for decades due to its special “non-absorbable” protein structure: lack of enzymatic pocket for conventional small molecules to bind; inaccessibility for antibody due to the predominant nucleus localization of c-Myc [44]. Previous studies have shown that HCK exerts a carcinogenic effect by regulating the ERK gene in BCR/ABL-positive ALL and B-ALL [13, 15]. ERK1/2 pathway have been identified in human myeloid leukaemia, and abnormal activation enhances the proliferation and survival of myeloid cells [45]. In our study, we considered that HCK might regulate CDK6 expression through the ERK1/2-c-Myc axis. However, we only conducted a simple research on the protein level, and further studies on the relationship between HCK and ERK1/2 were needed.

HCK is a member of the SFK family, including SRC, YES, LCK, FYN, FGR, BLK, LYN, YRK, and HCK [13]. Importantly, SFK members exhibit complementary and often functionally redundant roles in several signal transduction pathways. For example, FYN and LCK cooperate in T-cell receptor-mediated activation, and HCK and FGR cooperate to regulate macrophage phagocytosis [13, 34, 46]. This functional overlap between SFK members has been shown to play an essential role in maintaining relatively normal immune responses, which may be the cause that the effect of HCK on the self-renewal ability of LSCs is not fatal. Determining whether other SFK members are involved in maintaining the self-renewal of LSCs is an exciting and necessary future research direction.

Quiescent AML LSCs are not sensitive to chemotherapy and refractory to treatment [8]. Targeted therapies, such as FLT3 inhibitors, show promise in AML. In this study, HCK was expressed at higher levels on LSCs than HSCs and correlated with AML patient survival. The results are consistent with other researchers [19, 20]. Moreover, HCK is required for leukemogenesis and leukaemia maintenance, indicating that HCK may be an ideal therapeutic target for small molecule anti-AML compounds. In historical research, the addition of a special HCK inhibitor to AML therapy was shown to eliminate LSCs [20]. It has been a challenge to identify specific therapeutic targets of LSCs, because LSCs appear to use self-renewal signalling pathways shared by normal HSCs [28]. Therefore, the reasonable inhibitors that targeting LSCs is not only capable of alleviating leukaemia and enhancing the effect of chemotherapy, but is also able to maintain HSC regeneration. In our study, we found that HCK does not affect normal haematopoiesis or haematopoietic reconstruction but is critical for maintaining the self-renewal ability of LSCs by regulating the expression of CDK6. Therefore, HCK may be a potential therapeutic target. Unfortunately, we could not obtain specific HCK inhibitors, so we could not verify the effect of HCK inhibitors in the treatment of AML. We hope more specific HCK inhibitors appear in the future.

## Conclusions

In summary, we have provided several unexpected lines of evidence showing that HCK is highly enriched in LSCs and does not affect normal haematopoiesis, but that it is critical for maintaining the self-renewal of LSCs through the ERK1/2-c-Myc-CDK6 axis. Thus, HCK may be an ideal therapeutic target for eliminating LSCs with immune strategies.

## Supplementary Information

**Additional file 1:Supplemental Figure S1 **HCK gene-targeting strategy and PCR based identification.** (a)** HCK gene-targeting strategy for generating HCK^−/−^ and HCK ^fl/fl^, Rosa26-CreER mice. **(b)** PCR based identification.

**Additional file 2:Supplemental Figure S2 **HCK is highly expressed in AML cells. **(a)** The comparison of the level of HCK mRNA transcripts in AML cell lines with other different cell lines from the CCLE analysis. **(b)** Comparison of the expression level of HCK in AML patients and normal people.

**Additional file 3:Supplemental Figure S3** HCK is required for the proliferation of human AML cell lines.** (a)** U937 cells were transfected with shRNAs targeting HCK, followed by immunoblotting. **(b)** Representative images and numbers of colony formed by HCK-knockdown U937 cells. **(c)** Representative flow cytometric analysis of the cell cycle distribution of U937 cells transfected with targeted shHCK or SCR was determined using a BrdU incorporation assay. **(d)** MV4-11 cells were transfected with shRNAs targeting HCK, followed by immunoblotting. **(e)** Representative images and numbers of colony formed by HCK-knockdown MV4-11 cells after. **(f)** Representative flow cytometric analysis of the cell cycle distribution of MV4-11 cells transfected with targeted shHCK or SCR was determined using a BrdU incorporation assay.

**Additional file 4:Supplemental Figure S4 **HCK is not required for the proliferation of KG-1a cell lines.** (a)** KG-1a cells were transfected with shRNAs targeting HCK, followed by immunoblotting. **(b)** The numbers of KG-1a cells were counted at the indicated days after infection with shRNA targeting HCK or SCR. **(c)** Representative flow cytometric analysis of the cell cycle distribution in KG-1a cells transfected with shRNA targeting HCK or SCR was determined using a BrdU incorporation assay.

**Additional file 5:Supplemental Figure S5 **The first transplant to generate an MLL-AF9-driven leukaemia mouse.** (a)** The comparison of the appearance of MLL-AF9-driven leukaemia mice and normal C57BL/6 mice. **(b)** Representative images of spleens of MLL-AF9-driven leukaemia mice and normal C57BL/6 mice. **(c)** Histological H&E staining of the spleens in b. **(d)** Percent of YFP^+^ leukaemia cells in the peripheral blood at 36 days after the first transplantation. **(e)** Survival data for recipient mice receiving HCK^−/−^ or HCK^+/+^ YFP^+^c-Kit^+^ LSCs upon the first transplantation (n = 10; log-rank test). **(f)** No defect was found in the homing of HCK^−/−^ YFP^+^ leukaemia cells to the BM. **(g)** The level of HCK in mouse LSCs in the 2nd and 3rd transplantations. **(h)** The cell cycle distribution in YFP^+^cKit^+^ LSCs was determined using Ki-67 and Hoechst 33,342 staining in vitro.

**Additional file 6:Supplemental Figure S6 (a)** Potential candidates related to self-renewal and cell cycle were examined in HCK^+/+^ and HCK^−/−^ LSCs by RT-PCR. (b) Expression of SFKs in LSCs after deletion of HCK by RT-PCR.

**Additional file 7:** Supplemental **Table S1** Human patient samples used. **Supplemental Table S2 **Primer sequences for RT-PCR.

## Data Availability

The datasets generated/analysed during the current study are available.

## Abbreviations

LSCs: Leukaemia stem cells
AML: Acute myeloid leukaemia
HSC: Haematopoietic stem cells
HCK: Haematopoietic cell kinase
CDK6: Cyclin-dependent kinase 6
RT-PCR: Reverse transcription-polymerase chain reaction
BM: Bone marrow
BrdU: 5-bromodeoxyuridine
LT-HSC: Long-term haematopoietic stem cell
ST-HSC: Short-term haematopoietic stem cell
MPP: Multipotent progenitors
CMP: Common myeloid progenitors
GMP: Granulocyte-monocyte progenitors
MEP: Megakocyte-erythrocyte progenitors
CLP: Common lymphoid progenitors
TCGA: The Cancer Genome Atlas
