# Supplementary figures and images for: HCK maintains the self-renewal of leukaemia stem cells via CDK6 in AML
Source: J Exp Clin Cancer Res. 2021 Jun 24;40:210. doi: 10.1186/s13046-021-02007-4 (PMC8223385; doi:10.1186/s13046-021-02007-4)

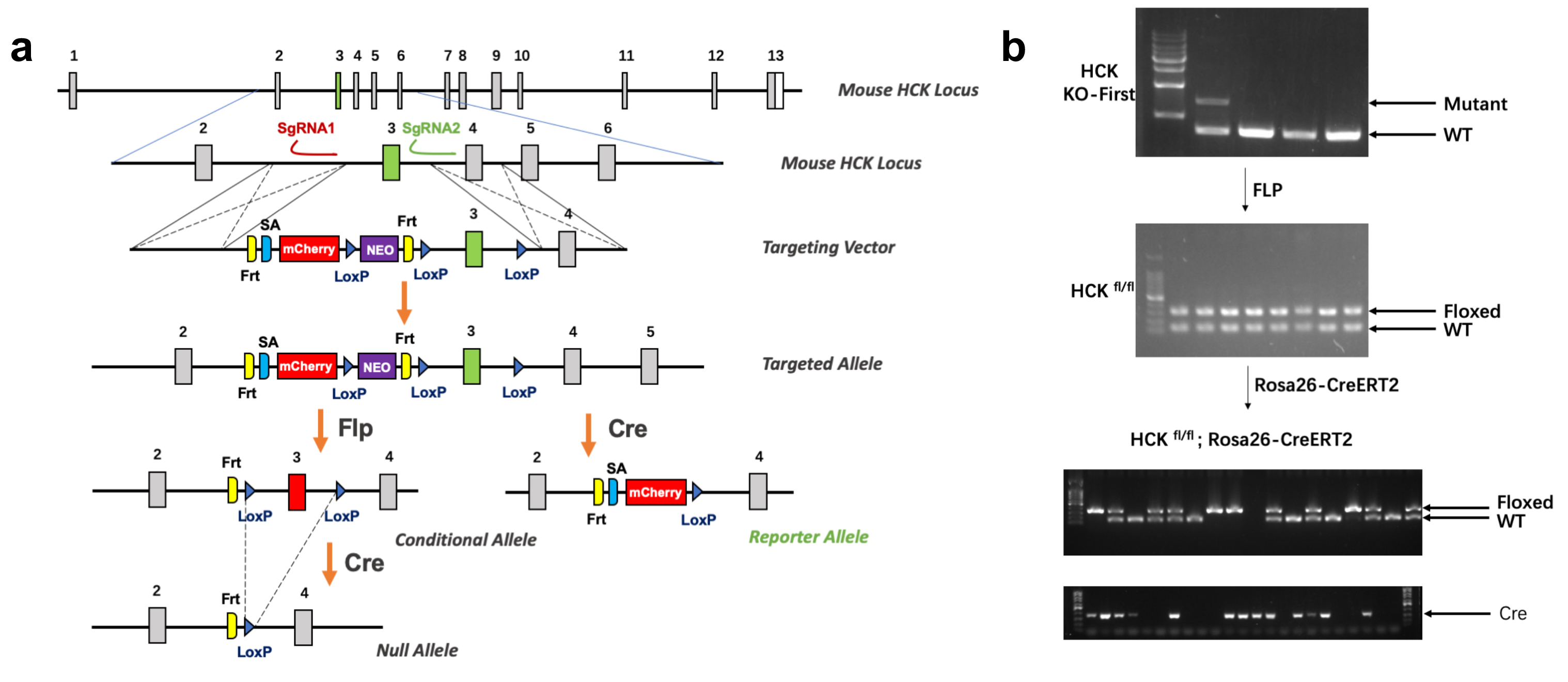

Supplement: Supplementary file 1 — Additional file 1:Supplemental Figure S1 HCK gene-targeting strategy and PCR based identification. (a) HCK gene-targeting strategy for generating HCK−/− and HCK fl/fl, Rosa26-CreER mice. (b) PCR based identification. [file 13046_2021_2007_MOESM1_ESM.png]

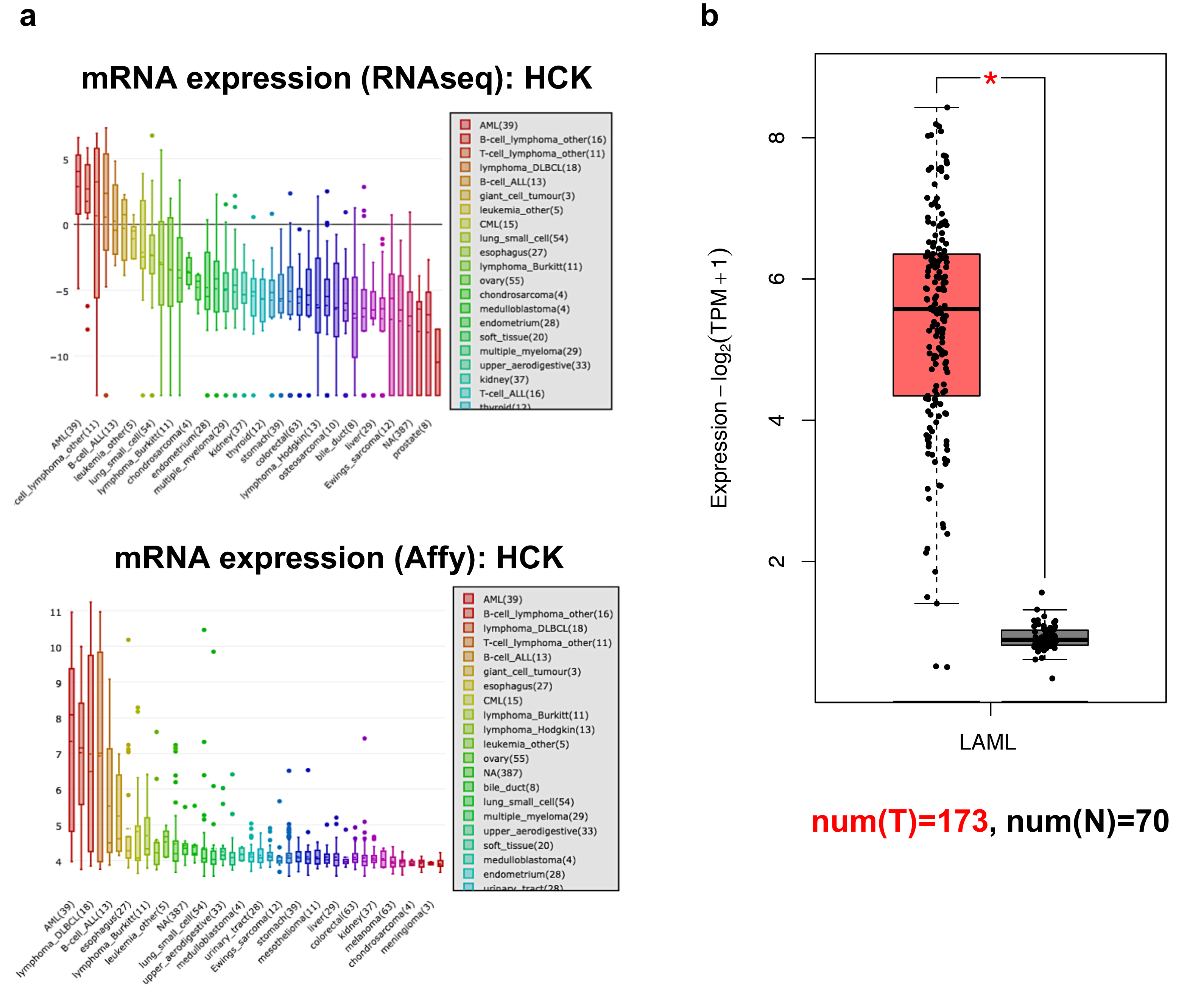

Supplement: Supplementary file 2 — Additional file 2:Supplemental Figure S2 HCK is highly expressed in AML cells. (a) The comparison of the level of HCK mRNA transcripts in AML cell lines with other different cell lines from the CCLE analysis. (b) Comparison of the expression level of HCK in AML patients and normal people. [file 13046_2021_2007_MOESM2_ESM.png]

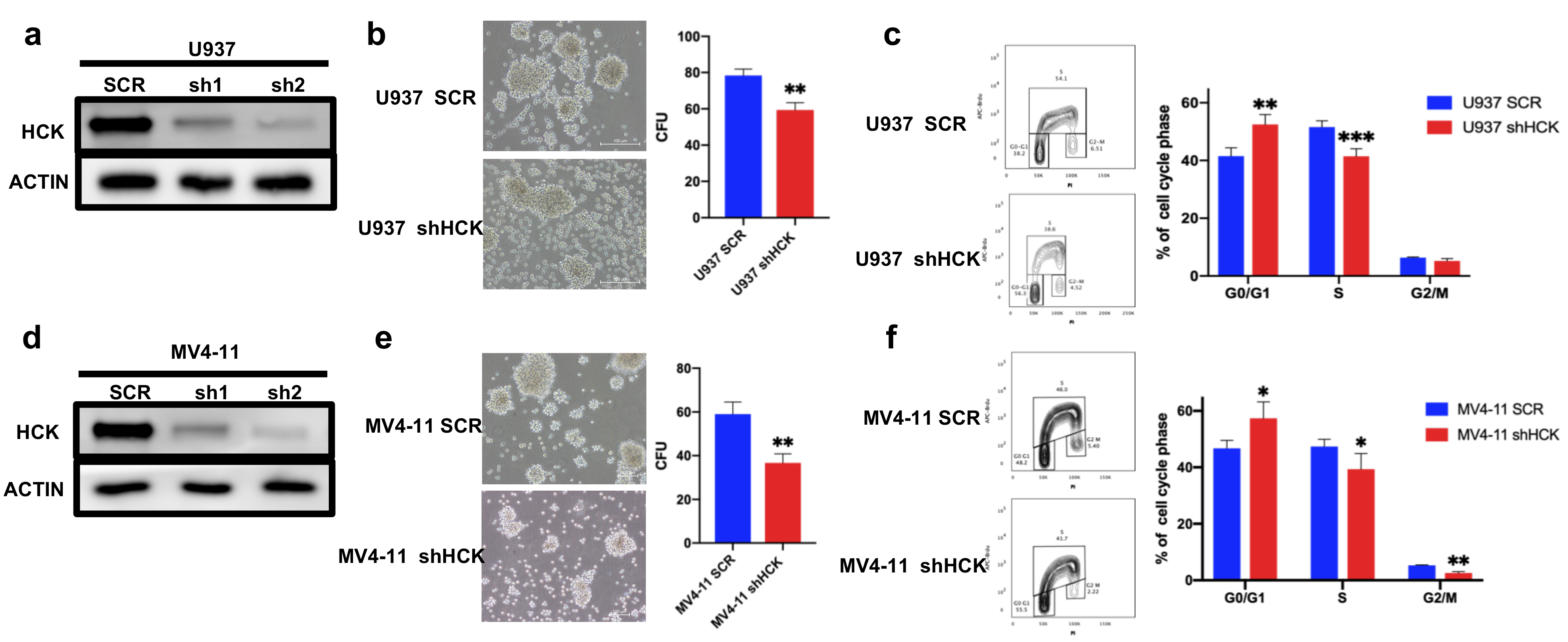

Supplement: Supplementary file 3 — Additional file 3:Supplemental Figure S3 HCK is required for the proliferation of human AML cell lines. (a) U937 cells were transfected with shRNAs targeting HCK, followed by immunoblotting. (b) Representative images and numbers of colony formed by HCK-knockdown U937 cells. (c) Representative flow cytometric analysis of the cell cycle distribution of U937 cells transfected with targeted shHCK or SCR was determined using a BrdU incorporation assay. (d) MV4-11 cells were transfected with shRNAs targeting HCK, followed by immunoblotting. (e) Representative images and numbers of colony formed by HCK-knockdown MV4-11 cells after. (f) Representative flow cytometric analysis of the cell cycle distribution of MV4-11 cells transfected with targeted shHCK or SCR was determined using a BrdU incorporation assay. [file 13046_2021_2007_MOESM3_ESM.png]

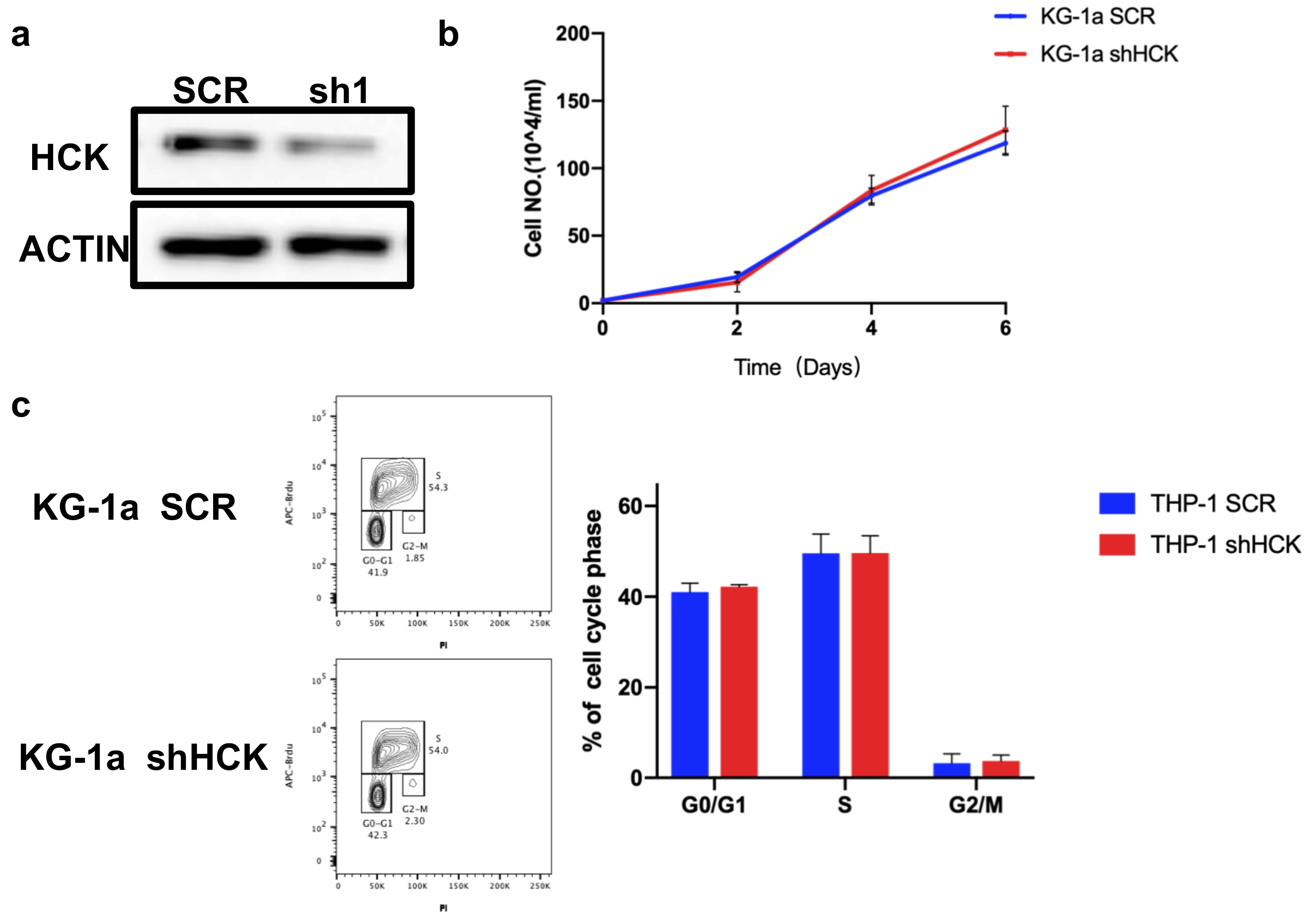

Supplement: Supplementary file 4 — Additional file 4:Supplemental Figure S4 HCK is not required for the proliferation of KG-1a cell lines. (a) KG-1a cells were transfected with shRNAs targeting HCK, followed by immunoblotting. (b) The numbers of KG-1a cells were counted at the indicated days after infection with shRNA targeting HCK or SCR. (c) Representative flow cytometric analysis of the cell cycle distribution in KG-1a cells transfected with shRNA targeting HCK or SCR was determined using a BrdU incorporation assay. [file 13046_2021_2007_MOESM4_ESM.png]

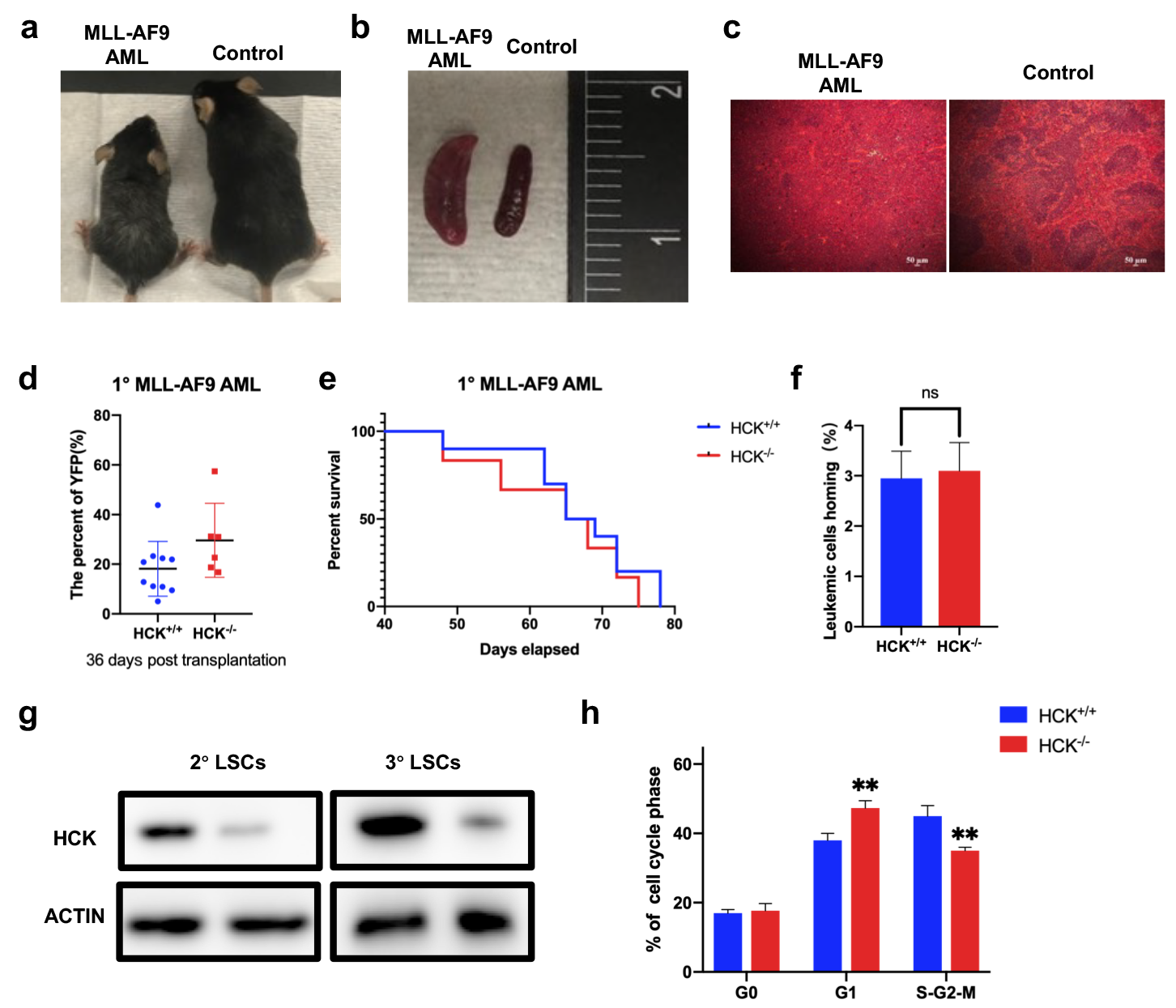

Supplement: Supplementary file 5 — Additional file 5:Supplemental Figure S5 The first transplant to generate an MLL-AF9-driven leukaemia mouse. (a) The comparison of the appearance of MLL-AF9-driven leukaemia mice and normal C57BL/6 mice. (b) Representative images of spleens of MLL-AF9-driven leukaemia mice and normal C57BL/6 mice. (c) Histological H&E staining of the spleens in b. (d) Percent of YFP+ leukaemia cells in the peripheral blood at 36 days after the first transplantation. (e) Survival data for recipient mice receiving HCK−/− or HCK+/+ YFP+c-Kit+ LSCs upon the first transplantation (n = 10; log-rank test). (f) No defect was found in the homing of HCK−/− YFP+ leukaemia cells to the BM. (g) The level of HCK in mouse LSCs in the 2nd and 3rd transplantations. (h) The cell cycle distribution in YFP+cKit+ LSCs was determined using Ki-67 and Hoechst 33,342 staining in vitro. [file 13046_2021_2007_MOESM5_ESM.png]

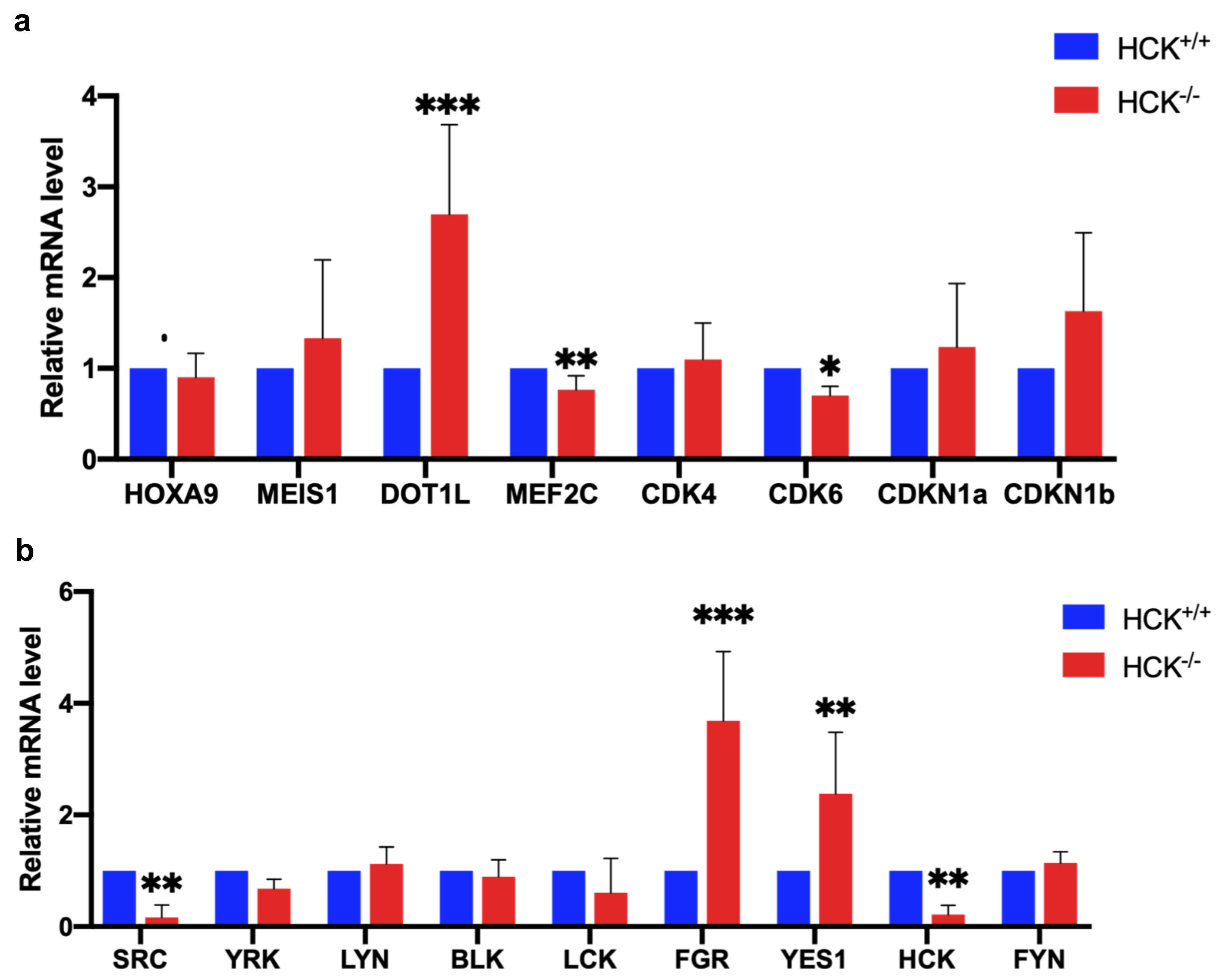

Supplement: Supplementary file 6 — Additional file 6:Supplemental Figure S6 (a) Potential candidates related to self-renewal and cell cycle were examined in HCK+/+ and HCK−/− LSCs by RT-PCR. (b) Expression of SFKs in LSCs after deletion of HCK by RT-PCR. [file 13046_2021_2007_MOESM6_ESM.png]
