# Supplementary material for: HCK maintains the self-renewal of leukaemia stem cells via CDK6 in AML
Source: J Exp Clin Cancer Res. 2021 Jun 24;40:210. doi: 10.1186/s13046-021-02007-4 (PMC8223385; doi:10.1186/s13046-021-02007-4)
Supplement: Supplementary file 7 — Additional file 7: Supplemental Table S1 Human patient samples used. Supplemental Table S2 Primer sequences for RT-PCR. [file 13046_2021_2007_MOESM7_ESM.docx]

﻿**Table 1. Human patient samples used**

|  | Gender | Age | Source | Disease |
| --- | --- | --- | --- | --- |
| Patient1 | Female | 70 | BM | AML-M4 |
| Patient2 | Male | 77 | BM | AML-M5 |
| Patient3 | Female | 57 | BM | AML-M2 |
| Patient4 | Male | 41 | BM | AML-M4 |
| Patient5 | Male | 26 | BM | AML-M2 |

**Table. 2 Primer sequences for RT-PCR**

| **Gene** | | **Sequence** | |
| --- | --- | --- | --- |
|  |  | **Forward (5′-3′)** | **Reverse (5′-3)** |
| **Mice** | **Actin** | GGCTGTATTCCCCTCCATCG | CCAGTTGGTAACAATGCCATGT |
|  | **Hck** | ﻿TCGTTGTCTGTTCGAGACTTTG | TCTTGTAGTGGAGCACGAGTT |
|  | **Cdk6** | TCTCACAGAGTAGTGCATCGT | CGAGGTAAGGGCCATCTGAAAA |
|  | **c-Myc** | CCCTATTTCATCTGCGACGAG | GAGAAGGACGTAGCGACCG |
|  | **CDK4** | ATGGCTGCCACTCGATATGAA | TCCTCCATTAGGAACTCTCACAC |
|  | **HOXA9** | AAAACACCAGACGCTGGAAC | CCAGGAGCGCATATACCTGC |
|  | **MEIS1** | TCAGCAAATCTAACTGACCAGC | AGCTACACTGTTGTCCAAGCC |
|  | **MEF2C** | GCCAGTTACCATCCCAGTGT | AACAGCACACAATCTTTGCCT |
|  | **CDKN1a** | CGGTGTCAGAGTCTAGGGGA | AGAGACAACGGCACACTTTG |
|  | **CDKN1b** | GGGTCTCAGGCAAACTCTGA | TCTGTTGGCCCTTTTGTTTT |
| **Human** | **ACTIN** | AGAGCTACGAGCTGCCTGAC | AGCACTGTGTTGGCGTACAG |
|  | **HCK** | AGGGCTACATCCCAAGCAAC | GGTCTCGCTATCCCGGATCA |
|  | **CDK6** | CCAGATGGCTCTAACCTCAGT | AACTTCCACGAAAAAGAGGCTT |
|  | **c-Myc** | GGCTCCTGGCAAAAGGTCA | CTGCGTAGTTGTGCTGATGT |

All sequences were obtained from PrimerBank (https://pga.mgh.harvard.edu/primerbank/).
